# The Notch Ligand Jagged1 as a Target for Anti-Tumor Therapy

**DOI:** 10.3389/fonc.2014.00254

**Published:** 2014-09-25

**Authors:** Demin Li, Massimo Masiero, Alison H. Banham, Adrian L. Harris

**Affiliations:** ^1^Radcliffe Department of Medicine, Nuffield Division of Clinical Laboratory Sciences, Weatherall Institute of Molecular Medicine, University of Oxford, Oxford, UK; ^2^Cancer Research UK Molecular Oncology Laboratories, Department of Oncology, Weatherall Institute of Molecular Medicine, University of Oxford, Oxford, UK

**Keywords:** Jagged1, Notch pathway, cancer therapy, cancer stem cells, angiogenesis

## Abstract

The Notch pathway is increasingly attracting attention as a source of therapeutic targets for cancer. Ligand-induced Notch signaling has been implicated in various aspects of cancer biology; as a consequence, pan-Notch inhibitors and therapeutic antibodies targeting one or more of the Notch receptors have been investigated for cancer therapy. Alternatively, Notch ligands provide attractive options for therapy in cancer treatment due to their more restricted expression and better-defined functions, as well as their low rate of mutations in cancer. One of the Notch ligands, Jagged1 (JAG1), is overexpressed in many cancer types, and plays an important role in several aspects of tumor biology. In fact, JAG1-stimulated Notch activation is directly implicated in tumor growth through maintaining cancer stem cell populations, promoting cell survival, inhibiting apoptosis, and driving cell proliferation and metastasis. In addition, JAG1 can indirectly affect cancer by influencing tumor microenvironment components such as tumor vasculature and immune cell infiltration. This article gives an overview of JAG1 and its role in tumor biology, and its potential as a therapeutic target.

Jagged1 (JAG1) is one of the five canonical ligands for Notch receptors expressed by mammalian cells. Along with Jagged2 (JAG2), it belongs to the Serrate/Jagged family, as opposed to the Delta/Delta-like family of ligands (DLL1, DLL3, and DLL4) (1, 2). JAG1, like the other canonical ligands, binds to Notch receptors and triggers activation, an interaction involving the Delta/Serrate/Lag-2 (DSL) domain of the JAG1 extracellular region (3, 4) (Figure 1). The JAG1 DSL domain is also responsible for its binding to the CD46 complement regulator, and this interaction is implicated in the functionality of T-helper cells (5). Of note, the binding and activating ability of JAG1 is tightly regulated by Notch receptor glycosylation (6–8). Recently, binding of the N-terminal C2 domain to phospholipid bilayers has been identified as a novel mechanism of modulating Notch activation induced by JAG1 (9). In addition, a number of publications indicate that the JAG1 intracellular domain can be released via γ-secretase-mediated cleavage, and the processed fragment, containing a PDZ-ligand motif at its C-terminus, has been reported to induce intrinsic reverse signaling within the ligand expressing cell (10–13). A soluble JAG1 extracellular domain, generated by ADAM17-mediated proteolytic cleavage, has also been implicated in mediating paracrine Notch signaling between endothelial cells and tumor cells (14), thus enabling Notch activation in more distant cells.

**Figure 1:**
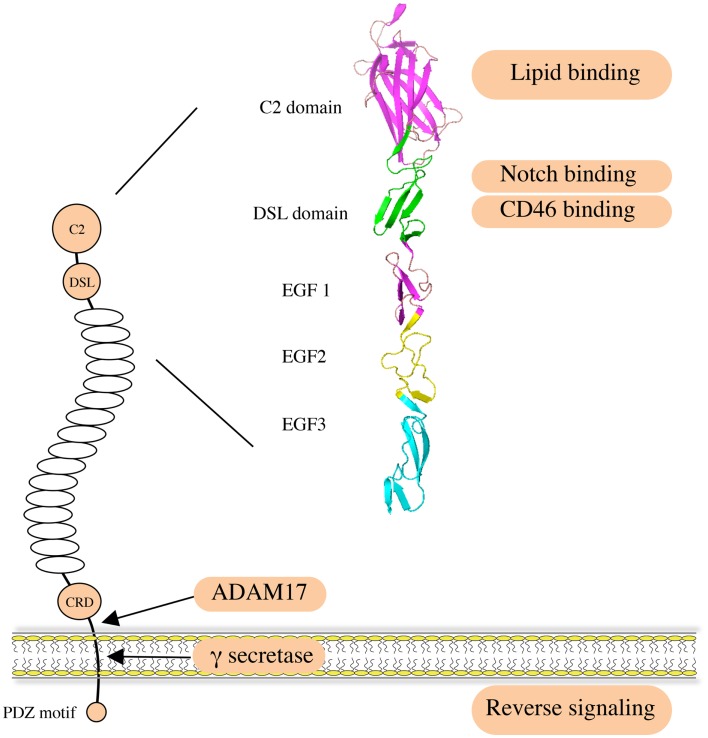
**Structure and function of human JAG1**. JAG1 is a type I transmembrane protein with an extracellular region featuring 16 epidermal growth factor (EGF) repeats followed by a cysteine-rich domain (CRD). Notch receptors bind to the DSL domain that is located N-terminal to the EGF repeats. A C2 phospholipid recognition domain at the very N-terminus reduces Notch activation upon binding to phospholipid bilayers. The CD46 recognition site largely overlaps with the Notch binding site. JAG1 can be cleaved by ADAM17 metalloprotease to release soluble protein, mediating paracrine Notch signaling on neighboring cells. It can also be processed intramembranously by γ-secretase to release the intracellular domain, within which the C-terminal PDZ-ligand motif is responsible for the intrinsic reverse signaling induced by JAG1.

Being a key component of the Notch signaling pathway, JAG1 plays an important role in both physiological and pathological conditions, including embryonic development and cancer. Among its physiological functions, it is worth noting that *Jag1* gene knockout in mice causes severe vascular defects that are lethal in early embryogenesis (15), and that JAG1 mutations in human beings are responsible for Alagille syndrome, an inherited multi-organ developmental disorder (16). In this review, we summarize what has been discovered about the contribution of JAG1 to tumor biology to date, and discuss the evidence supporting JAG1 as a valid target for cancer therapy.

## JAG1 Involvement in Cancer

**Figure 2:**
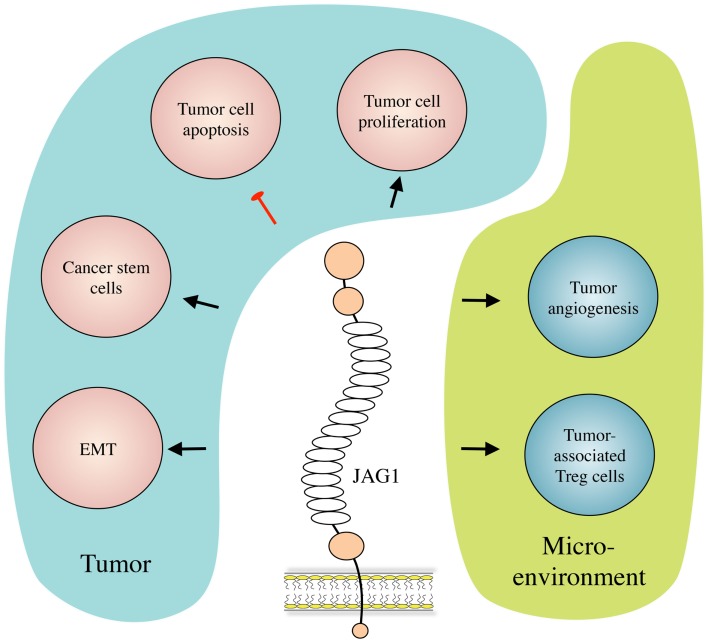
**JAG1 in cancer biology**. JAG1 expressed by cancer and/or stromal cells induces tumor cell growth and inhibits their apoptosis. JAG1 also induces and helps maintaining the cancer stem cell population, and enhances metastasis formation by inducing EMT. Meanwhile, in the tumor microenvironment, JAG1 promotes tumor-associated angiogenesis, and inhibits tumor-specific immunity by inducing regulatory T (Treg) cells.

Besides its role in Notch signaling in general (17), JAG1 has also been proven to play roles in multiple aspects of cancer biology, including tumor angiogenesis, neoplastic cell growth, cancer stem cells (CSCs), epithelial–mesenchymal transition (EMT), the metastatic process, and resistance to therapy in several types of cancer. Interestingly, JAG1 has been reported not only to be expressed and to play a role in cancer cells but its expression and activity have also been described in other cell types present in the tumor microenvironment such as mesothelial (18) and endothelial cells (14, 19), astrocytes (20), and osteoblasts (21). Importantly, JAG1 expression can be induced by other signaling pathways that are important in cancer such as TGF-β, WNT/β-catenin, IL-6, and NF-κB, as well as by the Notch pathway itself (22–26). We will first present and describe the mechanisms by which JAG1 exerts its functions in tumor biology (Figure 2), and then discuss its role in selected tumor types for which function and/or clinical relevance have been most extensively reported.

### Tumor angiogenesis

Angiogenesis refers to the growth of new blood vessels from existing ones, which is important in normal physiological processes such as embryonic development and wound healing. Angiogenesis also plays a key role in cancer biology, and it is recognized as one of the hallmarks of cancer (27, 28). Sprouting angiogenesis, the main mechanistic variant of this process, is initiated with endothelial tip cell invasion, followed by a series of maturation steps including lumen formation, and recruitment of perivascular cells. Notch ligands expressed on endothelial cells, and their cognate receptors on both endothelial and perivascular cells, smooth muscle cells, and pericytes, are involved in multiple stages of blood vessel formation from initial sprouting until vessel maturation (29, 30). DLL4 expressed by endothelial tip cells suppresses the tip phenotype in neighboring stalk cells, thus maintaining a sufficient number of endothelial cells for vascular integrity and adequate tissue perfusion (7). In some models, JAG1 has been proven to have the opposite effect in that it promotes endothelial cell proliferation and sprouting, and inhibits DLL4-induced Notch signaling in endothelial cells (7). Thus, JAG1 deletion inhibits sprouting angiogenesis, and JAG1 overexpression opposes DLL4 to promote sprouting.

JAG1 is also indispensable for vascular smooth muscle cell coverage of newly formed vessels, as well as in maintaining the interaction between endothelial cells and the perivascular cells. Endothelium-specific *Jag1* deletion causes deficits in vascular smooth muscle and fatal vascular defects (31). Endothelium-expressed JAG1 induces αvβ3 integrin expression, which in turn binds to VWF enriched on the basement membrane of the endothelial cells, facilitating smooth muscle adhesion, leading to vessel maturation. Genetic or pharmacologic disruption of JAG1, Notch, αvβ3, or VWF suppresses smooth muscle coverage of nascent vessels and arterial maturation during vascular development (32). The attachment of perivascular cells, such as smooth muscle cells, in turn regulates JAG1 expression and signaling through their surface-expressed Notch receptors. Perivascular cell-expressed Notch3 can also be induced by endothelial JAG1 and subsequently upregulates JAG1 expression on perivascular cells to form an autoregulatory loop that promotes both their maturation as pericytes and angiogenesis (33). Blockade of Notch signaling by knocking down Notch3 or JAG1 expression abrogates angiogenesis. This role during both the early and late stages of angiogenesis makes JAG1 the only Notch ligand for which such a broad function has been identified.

JAG1 has been reported to be strongly expressed by tumor-associated blood vessels, for example, in brain and ovarian cancer (34, 35), where it can trigger Notch signaling in tumor cells (angiocrine function) to promote tumor growth (19, 36, 37). Functionally, head and neck squamous cell carcinoma (HNSCC) tumors have been reported to secrete growth factors, such as HGF and TGF-α, that act in an autocrine/juxtacrine fashion to upregulate JAG1 expression through the MAKP pathway. This tumor-expressed JAG1 then stimulates endothelial cell sprouting, promoting angiogenesis and tumor growth in a mouse xenograft model (38). Furthermore, JAG1 expression levels also correlate with microvessel formation in human HNSCC tissues (38). A JAG1 pro-angiogenic role has also been reported in ovarian cancer models, where it was proven that Jag1 stromal silencing drastically reduced tumor microvascular density and neoplastic growth (39). All these points suggest that JAG1-targeted therapies could be of benefit to cancer patients even in the absence of tumoral JAG1 expression due to its role in tumor angiogenesis.

### Cancer stem cells

The Notch pathway plays an important role in normal stem cell biology, and this resulted in unacceptable levels of gastrointestinal toxicity when pan-Notch ablation was attempted using γ-secretase inhibitors (40, 41). Interestingly, unlike the Dll1/4 ligands, Jag1 was found to be dispensable for the homeostasis of normal intestinal stem cells (42), suggesting that JAG1 targeting is likely to have less side effects.

In the context of cancer, a small, distinct subpopulation of cells within tumors termed “cancer stem cells” (CSCs), tumorigenic cells, or cancer-initiating cells has been identified in several tumor types (43), including breast cancer (CD44^+^CD24^−/low^lineage^−^ and Aldefluor^+^ cell fractions), colon cancer (CD133^+^ cells), and cervical cancer (CD66^+^ cells). These cells are characterized by their self-renewal, high clonogenic potential, and asymmetric division producing daughter stem and differentiated cancer cells, enabling them to regenerate a tumor even when injected in very low numbers (44–51). CSCs are deemed to have increased invasive potential and resistance to several anti-cancer treatments and are often thought to be responsible for patient relapse and metastasis (52, 53). Therefore, therapies specifically targeting CSCs hold great potential for improving cancer treatment and outcome.

Notch signaling is important for both CSC maintenance and self-renewal (54, 55). Notch1 and Notch4 have been reported to have higher activity in the enriched breast CSC population, and inhibition of Notch signaling reduced stem cell activity *in vitro* and tumor formation *in vivo* (54, 56, 57). Similarly, Notch activity is 10- to 30-fold higher in the colon CSC population, where it has an important role in preventing apoptosis (58).

Several studies have functionally linked JAG1 to “stemness” in cancer, and it appears to be the main ligand driving CSC Notch signaling. In breast cancer, high levels of JAG1 promote stem cell self-renewal and potentiate mammosphere formation *in vitro* (23). In this context, JAG1 seems to play a central role in linking various pathways, involving well-established cancer-related molecules such as Notch3, interleukin-6 (IL-6), carbonic anhydrase IX (CAIX), and NF-κB (23, 26, 59, 60). JAG1 involvement in breast CSC has also been confirmed by mouse models in which mammary-specific deletion of *Lfng*, an N-acetylglucosamine transferase that prevents Notch activation by Jagged ligands, induces basal-like breast cancer with higher Jag1 activity and enhanced CSC proliferation (61). Likewise, JAG1 has also been involved in CSC biology in other tumor types. For example, JAG1 expressed by both tumor and endothelial cells plays an important role in glioma/glioblastoma-initiating cells (36, 37). Tumor-associated endothelial cell-expressed JAG1 has been also described to mediate lymphoma (19) and colon CSC maintenance. In the latter, soluble JAG1 produced by tumor-associated endothelial cells promoted the CSC phenotype in human colorectal cancer cells (14). Overall, these data indicate JAG1 as an important inducer of the stem cell phenotype in different cancer types and, importantly, demonstrates that both tumor and stromal JAG1 expression are relevant targets for CSCs.

### Epithelial–mesenchymal transition, invasion, and metastasis

The ability of tumor cells to invade the surrounding tissues and the ability of tumor cells to colonize distant organs (metastatic process) are both key features of aggressive cancers of pivotal clinical relevance. In order to escape their local environment, epithelial cells can exploit a reversible developmental program called EMT, during which loss of epithelial features (e.g., E-cadherin expression and cell-to-cell adhesion) and the acquisition of mesenchymal traits enables tumor cells to invade, resist apoptosis, disseminate and, as more recently observed, acquire stem cell features (28, 62–64). Notch signaling has been extensively studied in this context (55), and several reports have described JAG1 involvement in EMT, invasive potential, and metastasis particularly but not exclusively, in breast cancer. JAG1-induced signaling in breast cancer inhibits the epithelial phenotype via upregulation of the EMT master-regulator SLUG, and promotes tumor growth and metastasis (65). This is also reported to be important for TGFβ-induced EMT in mammary gland cells (22). Furthermore, JAG1 can increase tumor migratory and invasive behavior by inducing the urokinase-type plasminogen activator (uPA), a well-known marker of recurrence and metastasis (66). Finally, but not least, JAG1 has also been shown to be involved in the tissue specificity of breast cancer dissemination since it has been described to have significant roles in metastasis to the bone and brain (20, 21).

A role in EMT has also been described in cervical cancer, where JAG1 expression correlates with the rapid induction of phosphoinositol-3-kinase (PI3K)-mediated EMT (67); in hepatocellular carcinoma where it is repressed by the tumor-suppressor RUNX3 (68); and in treatment-resistant pancreatic cancer cells, where the JAG1-Notch2 axis controls several EMT transcription factors such as SNAIL, SLUG, and ZEB1 (69). Pro-invasive, migratory, and metastatic function has been also demonstrated for prostate cancer, where high JAG1 expression has been clinically linked to metastasis development and regulation of migration/invasion via NF-κB (70, 71), and for colon cancer, where it mediates APEX1 pro-tumorigenic functions and induces the metastasis markers MMP-2 and MMP-9 (72, 73). Overall, JAG1-induced Notch signaling appears to be implicated in different steps of the invasion/metastatic process. This, in conjunction with the relevance demonstrated in different tumor types, suggests JAG1 as an interesting therapeutic target to inhibit tumor cell invasiveness/metastasis.

### Tumor cell proliferation, cell cycle regulation, drug resistance, and survival

As for generalized Notch signaling, JAG1 can also directly affect more basic cellular functions such as cell cycle progression/proliferation and apoptosis/cell survival. In several cancer types, JAG1 induces tumor cell growth and promotes cell cycle progression. For example, it can directly regulate the cell cycle and induce proliferation by inducing cyclin D1 in breast cancer (74), cyclin D1, cyclin E, and c-Myc in colon cancer (73), or by promoting CDK2 kinase activity while repressing the p27 cell cycle suppressor in prostate cancer cells (75). Similar pro-proliferative functions have also been reported for glioma, myeloma, and ovarian cancer (18, 39, 76, 77). As anticipated, JAG1 can also affect cell death and there are several studies indicating that this Notch ligand exerts anti-apoptotic functions, although a mechanism remains to be fully elucidated. Interestingly, JAG1 can prevent both spontaneous apoptosis, for example, in glioma and prostate cancer cells (71, 77), as well as chemotherapy-induced cell death. Examples of the latter include lymphoma cells with respect to doxorubicin (19), ovarian cancer and taxanes (39), and pancreatic cancer and gemcitabine (69). While a role in spontaneous apoptosis prevention informs us regarding a physiological role played by JAG1, its role in chemo-protection represents a key finding as it highlights the opportunity to treat unresponsive tumor cases via combination chemotherapy treatments that include JAG1-Notch signaling blockade.

### T-cell regulation

A favorable tumor microenvironment is vital for cancer growth and survival. Various immune cells are found within the proximity of tumors, including T-cells, dendritic cells, macrophages, neutrophils, etc., but rarely the natural killer (NK) cells that are highly efficient in killing MHC downregulated tumor cells (78). Importantly, in many cancer types, there is an enrichment of T regulatory (Treg) cells capable of inhibiting tumor-specific immune responses and thus helping tumor cells evade immunosurveillance (79). Treg cells, in combination with other factors, such as myeloid-derived suppressor cells and cytokines, foster an immunosuppressive tumor microenvironment that supports tumor growth. Notably, inhibitors to immune checkpoints such as PD-1 and CTLA-4 can activate the tumor microenvironment and are showing exciting promise in the clinic in a variety of cancer types (78).

The induction and expansion of Treg cells in the tumor microenvironment involves crosstalk between tumor cells and dendritic cells, in which Notch signaling, and in particular JAG1-induced Notch activation, plays an important role. JAG1-Notch3 signaling has been reported to be essential for Treg induction and expansion stimulated by OX40L (80), whilst JAG1 expression in antigen presenting cells induces antigen-specific Tregs (81, 82). Furthermore, maturation of dendritic cells via JAG1 promotes survival and proliferation of Tregs (83).

JAG1-induced Notch signaling may also have impact on CD4^+^ T helper (Th) cell activation through a recently identified interaction between JAG1 and CD46. CD46 (MCP) is a ubiquitously expressed human type I transmembrane glycoprotein that was originally discovered as a complement regulatory protein and then a cell-entry receptor enabling viral infection. Activation of CD46 on CD4^+^ T-cells was shown to regulate the expression of Notch and its ligands, and, furthermore, JAG1 was identified as an additional physiological ligand for CD46 (5). The JAG1 binding site of CD46 overlaps with that of Notch receptors (5), and therefore, crosstalk between the complement and Notch systems may also be significant in cancer patients.

## JAG1 in Individual Cancer Types

There is a significant body of literature describing JAG1 functionality in a variety of tumor types, which is summarized in Table 1. The cancer types that have been most extensively studied are discussed in more detail below.

**Table 1:** **JAG1 involvement in individual cancer types**.

Tumor type	Oncogene or tumor suppressor	Observations	Reference
Breast cancer	Oncogene	Overexpression correlating with poor prognosis	(84, 85)
		Knockdown inhibiting cell growth, inducing cell cycle arrest	(74)
		Promoting cancer stem cells	(23, 26, 59)
		Promoting EMT via SLUG/E-cadherin	(65)
		Promoting osteolytic bone metastasis	(21)
		Association with triple negative/basal-like subtype	(74, 86–88)
Brain tumors	Oncogene	Promoting tumor growth	(37, 77)
		Maintaining cancer stem-like cells	(36)
		Overexpression in glioblastoma blood vessels	(35, 77)
Cervical cancer	Oncogene	Overexpression in cancer	(89)
		Downregulating Mfng	(89)
		Co-operating with HPV16-E6/E7 for cell transformation	(67)
		Downregulation suppressing cancer invasiveness	(90)
Colorectal cancer	Oncogene	Mediating Wnt-induced Notch activation in tumorigenesis	(24, 91, 92)
		Deletion reducing tumor growth in mouse model	(24)
		Mediating tumor invasion in mouse model	(93)
		Paracrine promotion of cancer stem cell phenotype – soluble JAG1	(14)
		Overexpression correlating with increased Notch activity in cancer	(73, 94)
		Tumor growth inhibition by JAG1 knockdown	(73)
		Mediating APEX1-induced cancer progression in mouse model	(72)
Endometrial cancer	Oncogene	Overexpression correlating with poor prognosis	(95)
Gastric cancer	Oncogene	Expression correlating with tumor aggressiveness and poor survival	(96)
		Tumor growth inhibition by JAG1 knockdown	(97)
Head and neck cancer	Oncogene	Notch/JAG1 co-expression indicating poor prognosis	(98)
Hepatocellular carcinoma	Oncogene	Overexpression correlates with tumor nodule number	(99)
		Maintaining cancer stem cells	(100)
		Overexpression correlating with poor outcome	(94, 101)
Non-small cell lung cancer	Oncogene	Silencing causing cancer cell apoptosis	(18)
		Preventing cancer cell apoptosis	(98, 102)
Ovarian cancer	Oncogene	Tumor-associated expression	(18, 34)
		Promoting cancer cell proliferation and dissemination via Notch3 activation	(18, 25)
		Knockdown impairing tumor growth and sensitizing to chemotherapy	(39)
Pancreatic cancer	Oncogene	Expression correlating with chemoresistance	(54, 69)
		Overexpression in tumors	(103)
		Expression associated with tumor angiogenesis	(104)
Prostate cancer	Oncogene	Upregulation in metastatic cases	(70)
		Knockdown reducing cell growth and invasion	(71, 75)
Renal cancer	Oncogene	Overexpression correlating with poor prognosis	(105)
		Inducing cell proliferation and adhesion	(106, 107)
Acute myeloid leukemia	Tumor suppressive	Suppressing cancer cell growth	(108)
		High expression correlating with favorable prognosis	(109)
	Oncogene	Driver of osteoblast mutated β-catenin-induced leukemogenesis	(110)
Anaplastic large cell lymphoma	Oncogene	Overexpression in tumor cells, promoting tumor cell proliferation and survival	(111)
B-Acute lymphoblastic leukemia	Oncogene	Supporting cancer cell survival	(112)
B-Chronic lymphocytic leukemia	Oncogene	Expression in tumor cells, inhibiting tumor cell apoptosis	(113)
Burkitt’s lymphoma	Oncogene	Angiocrine loop in vascular niche promoting tumor growth, aggressiveness, and chemoresistance	(19)
Hodgkin lymphoma	Oncogene	Overexpression in tumor cells, promoting tumor cell proliferation	(111)
Multiple myeloma	Oncogene	Overexpression in tumor cells, promoting tumor cell proliferation	(76, 114)

### Breast cancer

Multiple lines of evidence suggest that Notch signaling is involved in breast cancer development, maintenance and metastasis, and overexpression of Notch-1, -3, and -4 activated intracellular domains in mice causes aggressive and metastatic mammary tumors (115–119). Active forms of the Notch1 and Notch4 receptors also transform both normal human and murine mammary epithelial cells (116, 120, 121). When primary breast cancer samples are examined, accumulation of activated Notch1 and Notch3 is frequently observed in tumor cells (121, 122). Conversely, loss of a Notch negative regulator, Numb, is found to be associated with higher grade and worse prognosis in primary breast cancer (121, 123), and suppression of Notch activity by Notch3 and CSL knockdown promotes cancer cell apoptosis and inhibits tumor cell growth (122).

Unlike Notch pathway activation in T-cell acute lymphoblastic leukemia (T-ALL), which is mostly caused by *Notch1* gene mutations, the induction of Notch signaling in breast cancer (and other carcinomas) is predominantly associated with ligand-dependent mechanisms of activation. Studies indicate that JAG1 is the most prominent ligand involved in this aberrant Notch activation in breast cancer. JAG1 mRNA and protein are overexpressed in this tumor type, with high expression levels correlating with poor prognosis (84, 85). Functionally, *in vitro* JAG1 knockdown inhibits tumor cell growth inducing cell cycle arrest (74). JAG1-stimulated Notch signaling induces uPA, which is a validated marker of recurrence, high metastasic risk, and death from breast malignancy (66). JAG1 has a proven role in regulating breast CSC numbers (23, 26), EMT (65), and the metastatic process (21).

Two overlapping subtypes, triple negative (TN) breast cancer lacking estrogen receptor (ER), progesterone receptor (PR), and Her2 receptors, and basal-like breast cancer, which is generally associated with BRCA1 activation, are normally more aggressive and have poorer prognoses (124). These subtypes of breast cancers generally have higher levels of JAG1 expression, which correlate with reduced disease free survival (DFS). In contrast, the less aggressive luminal subtype, which is more associated with BRCA2 mutations, has lower JAG1 expression (74, 87, 88). In addition, a rare aggressive cell population in luminal-like cancers, lumino-basal cells, shares a gene signature with basal-like cancer, and their responsiveness to hormone therapy can be enhanced by blocking Notch signaling. Although a specific role for JAG1 was not investigated, these cells do have higher JAG1 expression than typical luminal-like cancers (125). These observations have also been confirmed by functional studies in breast cancer mouse models in which Jag1 played a key role in inducing a basal-like phenotype (61).

### Cervical cancer

Cervical cancer is the second most common cancer in women. Infection by high-risk human papillomavirus (HPV) such as HPV-16 and HPV-18, and the continued expression of viral oncoprotein E6 and E7 is linked to its development and progression (126).

The first indication for the involvement of Notch signaling in cervical cancer was the consistent pathway activation observed in this tumor type and in cervical metaplastic tissues but not in normal specimens (127). Furthermore, Notch1 expression correlates with disease progression, with little or no protein expression in normal cervical epithelium and high expression in precancerous and cancer tissues (128). Importantly, Notch1 protein is detected at high levels in the nucleus, indicating that activated Notch signaling may contribute to the progression of HPV-associated cervical neoplasia. Notch activation seems to contribute to cervical cancer development through its co-operation with HPV-16 E6 and E7 oncoproteins. Notch activation synergizes with HPV proteins in the transformation of immortalized human keratinocytes and primary keratinocytes (129, 130), and co-expression of the activated intracellular form of Notch1, along with HPV E6 and E7, can also support tumor growth *in vivo* (131).

Cervical cancer cells do not seem to present Notch-activating mutations, as sequencing of *Notch1* alleles failed to detect any of the mutations previously associated with T-ALL development (132). However, increased expression of the Notch ligand JAG1 was observed in cervical cancer cell lines and primary samples. JAG1 activity was indispensable for tumor maintenance as dominant negative JAG1 and RNA interference reduced cell line tumorigenicity *in vitro* (89). Overexpression of JAG1 in cervical cancer samples was coupled with the downregulation of Mfng, a negative regulator of the Jagged-Notch1 interaction (89). JAG1 also co-operates with HPV16-E6 and E7 oncoproteins in cell transformation and during *in vivo* tumor growth (67). The involvement of other Notch ligands in cervical cancer is unclear, but DLL1 expression was not upregulated in primary samples (89).

Targeting JAG1-induced Notch1 activation, by Notch1 RNA interference or by a γ-secretase inhibitor, suppressed cervical cancer invasiveness. Expression of microRNA-34a, which downregulates both Notch1 and JAG1, inhibits abnormal cell growth and suppresses cervical cancer invasiveness by repressing Notch-JAG1 signaling (90).

However, not all evidence supports a tumor-promoting role of Notch signaling in cervical cancer. Talora et al. found that Notch1 activity downregulation was required for sustained HPV-E6/E7 expression and subsequent steps of malignant transformation. Consequently, Notch1 activity was absent in their cohort of invasive cervical cancer samples (133). Notch1 activation has also been reported to induce the cervical cancer cell line HeLa to undergo apoptosis, growth arrest, and tumor growth suppression *in vivo* (134–136), although the role of JAG1 has not specifically been investigated within this tumor-suppressor context. The discrepancies between these results and the others could be due to the differences in sample cohorts, cell lines, and/or experimental reagents, and further studies are needed to reconcile the differences and to validate the role of Notch signaling in cervical cancer pathogenesis.

### Colon cancer

Colon cancer is one of the leading causes of cancer death worldwide, and the genetic causes of colon cancer involve mutations of oncogenes, suppressor genes, and multiple developmental pathways, including Wnt, Notch, Hedgehog, and BMP pathways. Mutations in the Wnt pathway cause colon cancer through constitutive activation of the β-catenin/TCF transcription factor complex (137). It has been demonstrated that pharmacological blockade of Notch activity using a γ-secretase inhibitor impairs intestinal homeostasis, and suppresses adenoma cell proliferation and induces differentiation, suggesting that tumorigenesis in this model requires a concerted activation of both Notch and Wnt (138). Further investigation proved that Notch works downstream of Wnt, as this pathway transcriptionally induces the Notch ligand JAG1 to trigger Notch activity (24, 91). Accordingly, deletion of the Wnt signaling inhibitor progastrin decreased JAG1 expression and Notch activation, and subsequently promoted the differentiation of colon cancer cells (92). Furthermore, deletion of JAG1 reduced tumor growth in the Apc^Min/+^ mouse model, confirming JAG1 as a pathological link between Wnt and Notch pathways in colon cancer (24). Stromal JAG1-induced Notch activation also mediated the tumor invasion and intravasation caused by the deletion of metastasis-suppressor gene Aes (Grg5) in Apc^Δ716^ intestinal polyposis mice, suggesting that JAG1-induced Notch signaling can be a promising target for prevention and treatment of colon cancer metastasis (93). Similarly, JAG1 expressed by endothelial cells has been implicated in fostering colorectal CSCs (14).

In human beings, Notch signaling was shown to be strongly activated in primary human colorectal cancers, and has an important role in cancer initiation and progression through the regulation of the main cellular functions associated with tumorigenesis, such as apoptosis, proliferation, angiogenesis, and cell migration. Microarray analysis discovered that the expression levels of *Notch1*, and its target *Hes1*, increased with increasing tumor grade (139). *In situ* hybridization on 130 colorectal cancer samples found that Notch signaling is constantly activated as measured by *Hes1* expression (86). Immunohistochemistry on a colon cancer tissue microarray confirmed that Hes1 is overexpressed in primary colon cancer tissues (140).

The cause of Notch overactivation in colon cancer appears to be ligand-dependent and to correlate with elevated JAG1 expression levels (73, 94). Accordingly, JAG1 knockdown leads to reduced Notch signaling activity that is accompanied by cell growth inhibition, cell cycle arrest, migration, and invasion inhibition, as well as tumor growth suppression (73). Apurinic-apyrimidinic endonuclease-1 (APEX1), a multiple-functional DNA repair enzyme, promotes colon cancer progression through activating the JAG1/Notch signaling pathway. APEX1-induced JAG1 expression in colon cancer cells, which subsequently activated Notch signaling to promote tumorigenicity, migration, invasion, angiogenesis, tumor formation, and metastasis in mouse xenograft models (72).

### Ovarian cancer

JAG1 is the main Notch ligand expressed by ovarian cancer cells; it is also strongly expressed by peritoneal mesothelial cells (18) and tumor-associated endothelial cells (34). JAG1 activates primarily Notch3 in ovarian cancer and promotes proliferation and dissemination within the intraperitoneal cavity (18). Its expression was induced by Notch3 activity itself, and was repressed by the WNT/β-catenin pathway (25). JAG1 gene silencing in tumor cells reduced viability and sensitized them to taxane treatment both *in vitro* and *in vivo*, where it drastically reduced tumor growth. Silencing of stromal-expressed *Jag1* also impaired tumor growth, without affecting tumor cell proliferation, by reducing the microvascular density. Combined tumor and stromal silencing proved to be synergistic, indicating the importance of targeting both the tumor and its microenvironment (39).

### Hematological cancers

The first oncogenic role for Notch signaling was described in T-ALL as a consequence of the identification of a high frequency of activating *Notch1* mutations (141–143). *Notch1* is also one of the most frequently mutated genes in chronic lymphocytic leukemia (CLL) (144, 145). In contrast to solid tumors, aberrations in hematological malignancies generally involve Notch receptors or pathway regulator mutations. While some mutations may enable ligand-independent pathway activation, others that stabilize the Notch intracellular domain (e.g., by targeting PEST motifs) still initially require their ligand-induced activation (143, 146).

Despite the predominance of genetic alterations as mechanisms of pathway alteration, Notch ligands such as JAG1 do play a part in some hematological cancers as they do in normal hematopoiesis (147, 148). Non-mutated Notch1 is highly expressed in Hodgkin lymphoma (HL) and anaplastic large cell lymphoma (ALCL) cells, and tumor-associated JAG1, overexpressed by bystander cells as well as by neighboring tumor cells, induces Notch1 activation and promotes tumor cell proliferation and survival (111). Similarly, in multiple myelomas, Notch1, Notch2, and JAG1 were highly expressed in primary tumor samples, and JAG1-induced Notch activation drove myeloma cell proliferation (76, 114).

Ligand-induced Notch overactivation was also observed in a subpopulation of primary B-CLL cells that are protected from spontaneous apoptosis as a result of JAG1 stimulation in *ex vivo* cultures (113). These cells overexpressed Notch ligands such as JAG1 and JAG2, and soluble JAG1 could stimulate Notch activation and increase B-CLL survival through the NF-κB pathway (113). Although this study was purely based on *ex vivo* systems, the results provide a good indication that JAG1 may play an important role in sustaining B-CLL cell survival.

Apart from inducing Notch activation from cancer cells themselves, JAG1 expressed by stromal cell also plays an important role in supporting cancer cell survival and tumor growth in hematological malignancies. B-ALL stroma express JAG1, JAG2, and DLL1, and these ligands are responsible for the synergistic activation of cancer cells that expressed Notch3 and Notch4, which ultimately support B-ALL cell survival (112). Interestingly, B-cell lymphomas have been reported to produce FGF4, which upregulates Jag1 on adjacent endothelial cells, that in turn induces Notch2 regulation of Hey1 in the lymphoma cells (19). This angiocrine FGF4-FGFR1/Jag1-Notch2 loop contributed to extranodal invasion and chemoresistance, thus extending the clinical relevance of Jag1 targeting into lymphomas that lack Notch receptor mutations or JAG1 expression.

Interestingly, Notch pathway activation does not always promote the pathogenesis of hematological malignancies. For example, Notch activation in acute myeloid leukemia (AML) suppressed, rather than promoted, cancer growth, whilst whole-genome expression analysis discovered that *Notch* signaling was silenced in AML (149, 150). *In vivo* and *ex vivo* activation of tumoral Notch, using genetic modification, induced cell cycle arrest, differentiation, and apoptosis in AML-initiating cells, and suppressed AML growth. JAG1 stimulation was also found to suppress AML cell line growth (108), and high JAG1 surface levels in leukemia cells was proposed as an independent favorable prognostic factor in AML patients (109). These results suggest that promoting JAG1-stimulated Notch activity could be a potential route for novel therapy in AML. However, further studies are required; first, as the expression level of the Notch1 ICD did not correlate with AML outcome (109), and second, a separate study reported activating mutations of β-catenin in mouse osteoblasts that induced AML development through Jag1-induced Notch activation (110). This group reported that a large proportion (38%) of patients with myelodysplastic syndromes or AML had osteoblast associated β-catenin activation-induced Notch signaling (110).

## Therapeutic Potential and Implications

It is well established that the Notch pathway (17), and in particular JAG1-induced Notch activation, plays important roles in tumor biology, affecting both cancer cells and multiple components of the neoplastic microenvironment (e.g., vasculature and immune cells). This, in addition to the facts that JAG1 is often upregulated in tumor cells (although generally not mutated in cancer), and that ligand-induced activation is required even in the presence of some Notch receptor mutations, makes it a particularly attractive target for therapy (143).

Generally, the main concern in targeting the Notch pathway by pan-Notch inhibitors was the resulting gastrointestinal toxicity (41). Antibodies targeting individual Notch receptors (151, 152) seem to avoid toxicity but, in some situations, tumors may have high levels of more than one receptor (e.g., Notch1 and Notch2 in CLL, Notch1 and Notch4 in breast CSCs), or potentially, the roles of individual Notch receptors may contribute differently in the tumor versus its microenvironment, which adds further complications. For example, while Notch1 is often oncogenic when expressed by cancer cells, Notch3 expressed by perivascular cells plays a major role in JAG1-mediated vascular function (33), and it is also important in Treg induction and expansion in the tumor microenvironment (80).

JAG1 has a number of advantages as a target for anti-cancer therapy over other Notch ligands. DLL4 dysfunction exhibited haploinsufficiency (153), while that of JAG1 did not (15). Thus, targeting JAG1 may provide a greater therapeutic window in which to reduce Notch activity without causing severe adverse effects. In addition, while in the context of cancer, DLL4 mainly functions in the vasculature, JAG1 has roles in vasculature, immunosuppressive Treg cells, and the tumor/stem cells. Thus, targeting JAG1 on both stroma and tumor cells could induce synergistic effects as demonstrated in an ovarian cancer model (39). The fact that chronic blockade of DLL4 causes severe pathological changes in animal models (154) makes alternative targets such as JAG1 even more appealing.

Crosstalk between Notch signaling and other pathways, such as Wnt, Hedgehog, and vascular endothelial growth factor (VEGF) pathways, as well as with the immune system, make Notch and its ligands attractive approaches for combination therapy. Bevacizumab treatment targeting VEGF-induced angiogenesis has made a profound impact on various cancer types, but resistance to treatment is frequently observed in both preclinical and clinical settings (155). Increased pericyte coverage of the tumor vasculature that maintains its integrity is thought to be one of the main mechanisms that cause resistance to anti-angiogenic treatments (156). Therefore, the role of endothelium-expressed JAG1 in vascular smooth muscle biology (32) suggests that targeting JAG1 may have beneficial synergistic effects with anti-angiogenic approaches.

Furthermore, due to its anti-apoptotic and pro-“stemness” functions, JAG1 blockade represents an attractive option also for combination therapy approaches with standard chemotherapy as demonstrated in preclinical models of ovarian and pancreatic cancer and lymphoma (19, 39, 69).

In conclusion, targeting JAG1 will provide a new approach to act on multiple aspects of tumor biology, and represents a promising new strategy in developing novel anti-tumor therapies.

## Conflict of Interest Statement

The authors are inventors and contributors of a patent on Jagged1 therapeutic antibody development.
